# Structural Basis of Potential Inhibitors Targeting SARS-CoV-2 Main Protease

**DOI:** 10.3389/fchem.2021.622898

**Published:** 2021-03-12

**Authors:** Hylemariam Mihiretie Mengist, Tebelay Dilnessa, Tengchuan Jin

**Affiliations:** ^1^ Department of Obstetrics and Gynecology, The First Affiliated Hospital of USTC, Division of Life Sciences and Medicine, University of Science and Technology of China, Hefei, China; ^2^ Hefei National Laboratory for Physical Sciences at Microscale, CAS Key Laboratory of innate immunity and chronic disease, School of Basic Medical Sciences, Division of Life Sciences and Medicine, University of Science and Technology of China, Hefei, China; ^3^ Department of Medical Laboratory Science, College of Health Science, Debre Markos University, Debre Markos, Ethiopia; ^4^ CAS Center for Excellence in Molecular Cell Science, Chinese Academy of Science, Shanghai, China

**Keywords:** COVID-19, SARS-CoV-2, crystal structure, main protease, inhibitors

## Abstract

The Coronavirus disease-19 (COVID-19) pandemic is still devastating the world causing significant social, economic, and political chaos. Corresponding to the absence of globally approved antiviral drugs for treatment and vaccines for controlling the pandemic, the number of cases and/or mortalities are still rising. Current patient management relies on supportive treatment and the use of repurposed drugs as an indispensable option. Of a crucial role in the viral life cycle, ongoing studies are looking for potential inhibitors to the main protease (M^pro^) of severe acute respiratory syndrome Coronavirus -2 (SARS-CoV-2) to tackle the pandemic. Although promising results have been achieved in searching for drugs inhibiting the M^pro^, work remains to be done on designing structure-based improved drugs. This review discusses the structural basis of potential inhibitors targeting SARS-CoV-2 M^pro^, identifies gaps, and provides future directions. Further, compounds with potential M^pro^ based antiviral activity are highlighted.

## Introduction

Since its emergence in Wuhan, China (Huang et al., 2020), COVID-19 (caused by the novel SARS-CoV-2) has been causing significant mortality and morbidity worldwide. The pandemic sparked global attention affecting every corner of the world and is changing the social, economic, and political status of the globe. As of 15 December 2020, the number of confirmed cases is over 73 million and deaths have surpassed 1.63 million (https://www.worldometers.info/coronavirus/). Curbing the spread of the virus has been challenging as it has various means of transmission including direct contact, *via* droplets, airborne, fomite, fecal-oral, bloodborne, sexual intercourse, ocular, mother-to-child, and animal-to-human (Patel et al., 2020). Although the virus primarily causes a mild respiratory illness, significant proportions of patients experience severe disease with outcomes of death. Moreover, there is also a significant number of asymptomatic infections that can transmit the virus to others. COVID-19 patients with underlying conditions are known to have a higher risk of developing a severe disease (Chow et al., 2020; Zhang et al., 2020c).

The Remdesivir drug and the Pfizer vaccine have been approved by the USA FDA for emergency use, but there are (at the time of writing this article) no globally approved specific antiviral drugs and vaccines for official use. The primary treatment relies on symptomatic and oxygen therapy to manage respiratory impairment. When there is respiratory failure, mechanical ventilation is recommended to prevent respiratory arrest. In the case of complicated disease, intensive care is needed because of acute respiratory distress syndrome (ARDS) or multiple organ failure (MOF) (Cascella et al., 2020; Chen et al., 2020a; Gattinoni et al., 2020). Fifteen drugs (chloroquine, hydroxychloroquine, lopinavir, ritonavir, nafamostat, camostat, famotidine, umifenovir, nitazoxanide, ivermectin, corticosteroids, tocilizumab, sarilumab, bevacizumab, and fluvoxamine) are under clinical trial but conducting solid clinical trials is reportedly more difficult with increased public inquiry over readily available drugs (Shaffer, 2020). A combination of drugs could be more effective; for example, a combination of antitussive noscapine and hydroxychloroquine showed a strong binding affinity to SARS-CoV-2 M^pro^ (Kumar et al., 2020b). A tremendous number of studies are underway to determine the therapeutic use of antivirals (bemcentinib, chloroquine & hydroxychloroquine, lopinavir boosted with ritonavir and remdesivir) and immune modulators (anakinra and canakinumab, azithromycin, brensocatib, convalescent plasma, corticosteroids, interferon beta, ruxolitinib, mesenchymal stromal cells and sarilumab and tocilizumab) to treat COVID-19 (Connelly, 2020).

Treatment of COVID-19 is medically unmet and designing potential drugs that could halt infection and disease progression is critical. Designing drugs that directly act on conserved enzymes like the main protease or 3C-like protease (M^pro^ or 3CL^pro^), papain-like protease (PL^pro^), non-structural protein 12 (nsp12), and RNA-dependent RNA polymerase (RdRP) could be broad-spectrum and effective (Zumla et al., 2016). Remdesivir is one of the antivirals under clinical trial for COVID-19 treatment with probable inhibition of RNA synthesis *via* targeting RdRP (Saha et al., 2020). In a randomized controlled trial of 1,062 patients, compared to a placebo, remdesivir significantly shortened the recovery time of adult COVID-19 patients, suggesting its therapeutic role (Beigel et al., 2020). Its clinical effect on severely ill patients, however, is controversial.

Several studies combining structure-based, virtual, and high-throughput screening methods are currently underway to identify SARS-CoV-2 M^pro^ inhibitors (Zhu et al., 2020). Summarizing the results of these studies, identifying their gaps, and appraising critiques are crucial to putting forward strong recommendations and future directions. Therefore, this review discusses recent advancements and prospects of structure-based drug designing activities that target SARS-CoV-2 M^pro^.

## SARS-CoV-2 M^pro^ as a Drug Target

For many viruses, the protease enzyme plays a critical role in viral protein maturation by cleaning proproteins after their translation into the host cell cytosol. As a result, viral proteases are often potential drug targets. The inhibition of viral protease can reduce the assembly of mature viral particles. To date, many antiviral drugs have been developed against viral infections *via* targeting proteases. For instance, HIV-1 protease inhibitors (tipranavir, darunavir, amprenavir, lopinavir, saquinavir, atazanavir, indinavir, ritonavir, and nelfinavir) (Lv et al., 2015) and hepatitis C virus (HCV) NS3/4A protease inhibitors (boceprevir, telaprevir, ritonavir, asunaprevir, paritaprevir, grazoprevir, glecaprevir, voxilaprevir, and sofobuvir) (de Leuw and Stephan, 2017) are amongst the FDA approved drugs. Therefore, formulating antiviral drugs inhibiting SARS-CoV-2 M^pro^ could also have potential clinical use.

**FIGURE 1:**
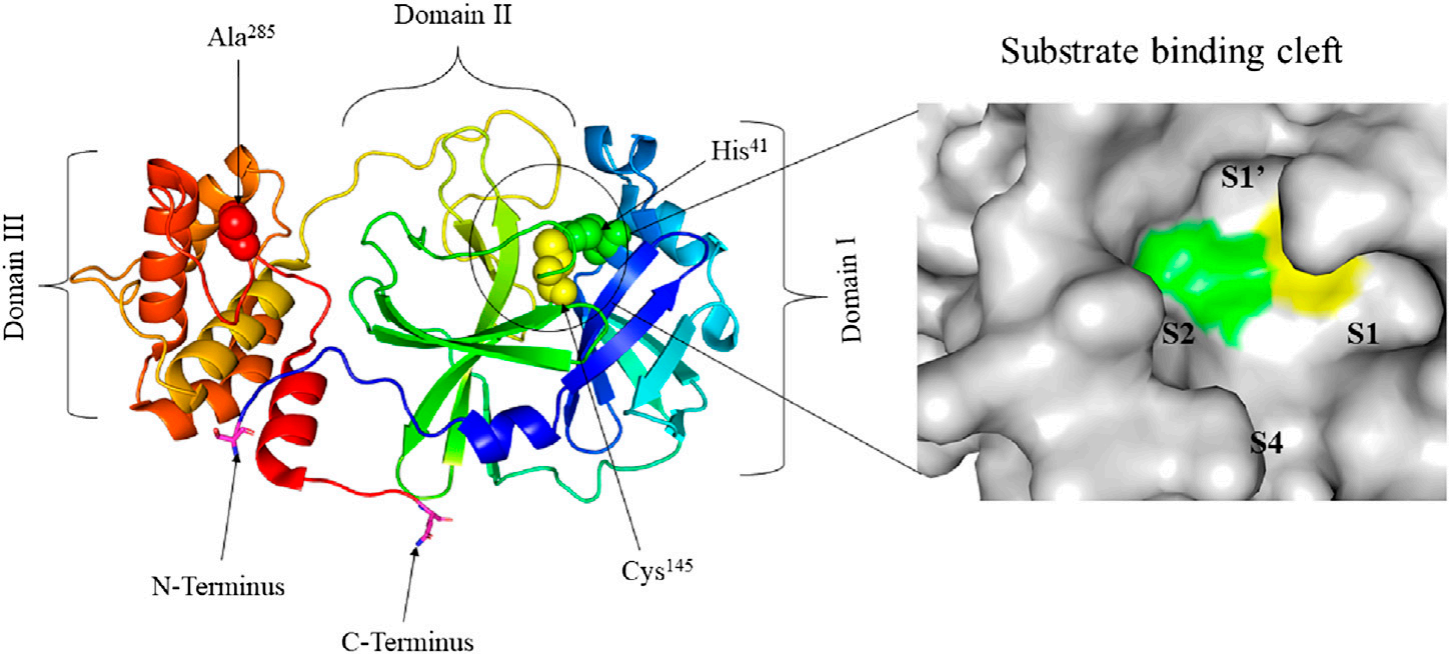
Crystal structure of free SARS-CoV-2 M^pro^ solved at 1.75 Å resolution (PDB entry: 6Y2E (Zhang, et al., 2020a)) **(left)** and surface view of the substrate-binding cleft **(right)**. The three distinct domains of the protomer are indicated. His^41^ (green) and Cys^145^ (yellow) residues of the catalytic dyad and Ala^285^ (red) of Domain III are represented in spheres. The substrate-binding cleft between Domains I and II is encircled. Ser^1^ of N-terminus and Gln^306^ of C-terminus are represented in sticks and their carbon atom is highlighted in magneta. The four subsites of the substrate-binding cleft are indicated.

SARS-COV-2 is one of the seven medically important coronaviruses and has been causing the most catastrophic once in a century disease of pathogens (Cui et al., 2019; Gates, 2020; Gorbalenya et al., 2020). SARS-CoV-2 is an enveloped betacoronavirus with a positive-strand large RNA genome (Holmes and Lai, 1996; Fehr and Perlman, 2015; Lu et al., 2020). Although SARS-CoV-2 has a large RNA genome of about 30 kb, it encodes only a few proteins (Dömling and Gao, 2020). Among these proteins, M^pro^, a cysteine protease, mediates the maturation cleavage of polyproteins during virus replication (Gorbalenya et al., 1989; Lee et al., 1991; Ziebuhr et al., 2000). The M^pro^ is a homodimer containing two protomers each, comprising three domains (Domains I, II, and III). Domains I and II, comprised of residues 8–101 and 102–184, respectively, are made up of six antiparallel β-barrels. An antiparallel globular cluster of five α helices forms domain III (residues 201–303) which is connected to domain II *via* a long loop region (residues 185–200). In the cleft between domains I and II, there is a Cys-His catalytic dyad which, together with N-terminus residues 1 to 7, is thought to have a vital role in proteolytic activity (Anand et al., 2002; Anand et al., 2003; Yang et al., 2003; Shi et al., 2004; Xue et al., 2007). The substrate-binding site is located in the cleft between domains I and II and the protomers, which bind each other through N-terminus residues 1-7, are located between domains II and III with roles in the formation of the substrate-binding site (Yang et al., 2003; Chou et al., 2004; Hsu et al., 2005; ul Qamar et al., 2020; Zhang et al., 2020a). The substrate-binding cleft is comprised of four subsites namely; S1’, S1, S2, and S4 (Figure 1). The M^pro^ is a conserved protein across all coronaviruses and the amino acids in substrates are numbered as -P4-P3-P2-P1 and P1’-P2’-P3’- from the N-terminus to the C-terminus (Hayden et al., 2003; Yang et al., 2005; Kim et al., 2016). The cleavage site is located between P1 and P1’ and a glutamine residue is required in the P1 position (Dai et al., 2020; Jin et al., 2020a; Su et al., 2020; Zhang et al., 2020a).

The interaction between the two protomers determines the activity of the enzyme. The interaction of the N-terminus of one protomer with domain II of the other *via* hydrogen bonding, helps shape the S1 pocket of the active site. Therefore, the dimer is the active form while the monomer is inactive. Dimerization involves intermolecular interactions between the two protomers. Salt bridges between the N-terminus domain III of one protomer and electrostatic and hydrophobic interactions between two different domains III help enzyme dimerization (Fan et al., 2004; Zhang et al., 2020a). Grottesi et al. (2020) used computational approaches to study the structure and function of SARS-CoV-2 M^pro^. The authors demonstrated that when the average volume of the binding pocket increases in one chain, a decrease takes place in the other chain. Moreover, the interactions between the N-terminus and domain III of one monomer stabilizes the residues in the pocket. While dimerization is crucial for enzyme activity, one protomer is active and the other is inactive.

Understanding the atomic-level mechanism of the peptide cleavage, catalyzed by cysteine proteases, is crucial for designing structure-based potent inhibitors. Earlier studies proposed that the imidazole group of histidine polarizes and activates the SH group of the cysteine forming CysS^−^/HisH^+^ ion which has a high nucleophilic property that reacts with substrates (Keillor and Brown, 1992). A QM/MM study reported that proteolysis catalyzed by cruzain cysteine protease has acetylation and deacetylation stages. In the acetylation step, cysteine attacks the carbonyl carbon atom of the peptide after which the proton from the protonated HisH^+^ is transferred to the nitrogen atom of the scissile peptide bond. Then the deacetylation stage is supposed to be assisted by a water molecule activated by histidine (Arafet et al., 2017; Swiderek and Moliner, 2020).

A similar study by Swiderek and Moliner (2020) presented the cleavage of a polypeptide, Ac-Val-Lys-Leu-Gln-ACC, catalyzed by SARS-CoV-2 M^pro^. First, a proton is transferred from Cys^145^ to His^41^ with a simultaneous nucleophilic attack of the carbonyl carbon atom of the peptide bond by the sulfur atom of Cys^145^ which results in a thiohemiketal intermediate. The transfer of a proton from His^41^ to the nitrogen atom of the substrate, which forms an acyl-enzyme complex intermediate, assists the cleavage of the peptide bonds. This reaction produces the first product ACC released from the active site. After the release of ACC, an activated water molecule attacks the carbonyl carbon atom of Gln^5^ of the peptide with simultaneous transfer of proton to His^41^. Finally, the second product species is released after the covalent bond between Cys^145^ and the peptide in the thiohemiketal intermediate is broken.

A DFM/MM simulation study revealing an equivalent mechanism of the reaction is reported by Ramos-Guzmán et al. (2020) using the peptidomimetic Ac-Ser-Ala-Val-Leu-His-aldehyde inhibitor as a substrate. These studies provide insights into the structure-based design of potential M^pro^ inhibitors that can form a stable enzyme-inhibitor complex similar to the product in the acetylation step of the proteolysis reaction. These studies help the scientific community deeply understand the structure and function of the M^pro^ which is important for effectively designing potent inhibitors.

The main protease of coronaviruses is a potential drug target since it is responsible for the maturation of itself and other important polyproteins (Ziebuhr et al., 2000). SARS-CoV-2 has 14 open reading frames (ORFs). The M^pro^ (nsp5), encoded by the major ORF1ab, cleaves two overlapping polyproteins (pp1a and pp1ab) into 16 non-structural proteins which are important for viral replication and maturation (Paul, 2006; Ziebuhr et al., 2000; Chen et al., 2020b; Gordon et al., 2020). In addition, it plays a significant role in virus entry to host cells where inhibition of this enzyme halts the viral entry and the subsequent infection (Jain and Mujwar, 2020). These important functions of the viral protease enzyme purpose itself are an interesting therapeutic target for curbing coronavirus associated diseases (Thiel et al., 2003; Naqvi et al., 2020). Structurally optimized broad-spectrum drugs are effectively inhibiting the main protease of coronaviruses pertaining to its relatively conserved nature (Yang et al., 2005; Xue et al., 2007). Similarly, SARS-CoV-2 M^pro^ has attracted great attention to the development of drugs to fight the ongoing COVID-19 pandemic. In addition to the M^pro^, other proteins including spike protein (S), RdRP, NTPase/helicase, and papain-like protease are currently alternative drug targets (Wu et al., 2020). The M^pro^ is also critically important for the proteolytic release of enzymes essential for viral replication including nsp 13 which has NTPase and RNA helicase activity (Thiel et al., 2003; Shu et al., 2020). Homology modeling studies presented structural similarity between SARS-CoV-2, SARS-CoV, and MERS-CoV main proteases with a conserved active site (Stoermer, 2020; Ullrich and Nitsche, 2020), and it is also noted that the binding of lead compounds is similar in both SARS-CoV and SARS-CoV-2 main proteases regardless of the protonation state of the Cys-His catalytic dyad (Macchiagodena et al., 2020) indicating the possibility of designing broad-spectrum drugs against these viruses.

The enhanced activity observed in SARS-CoV-2 M^pro^ providing evidence for repurposing it as a potential drug target. Mutations (Ser284Ala, Thr285Ala, and Ile286Ala) in SARS-CoV M^pro^ are reported to result in an enhanced activity where the two similar mutations (Thr285Ala and Ile286Leu) in SARS-CoV-2 M^pro^ caused the higher activity of SARS-CoV-2 M^pro^ over SARS-CoV M^pro^ (Lim et al., 2014; Zhang et al., 2020a). Thus, the design of improved broad-spectrum inhibitors should consider key amino acid differences that occur in SARS-CoV-2 M^pro^ compared to previous viruses. Main proteases also have substrate recognition site preference in cleaving polyproteins which is important when designing specific inhibitors. The main proteases of the three viruses (SARS-CoV-2, SARS-CoV, and MERS) have very similar substrate recognition profiles with heightened preference to glutamine in the P1 of polyproteins, but SARS-CoV M^pro^ demonstrated broader substrate specificity at the P2 position given that the two enzymes (SARS-CoV and SARS-CoV-2 M^pro^) prefer leucine at this position (Rut et al., 2020a). Interestingly, human host-cell proteases with such similar specificity have not been reported yet causing the anticipated drugs to have reduced off-target activities (Hilgenfeld, 2014; Zhang et al., 2020b) proving that is was the right decision to select SARS-CoV-2 M^pro^ as an outstanding drug target. Therefore, although it needs clinical evidence, M^pro^ targeting drugs are thought to be suitable for human beings and have fewer side effects.

## Structure-based Design of Drugs That Target SARS-CoV-2 M^pro^

**FIGURE 2:**
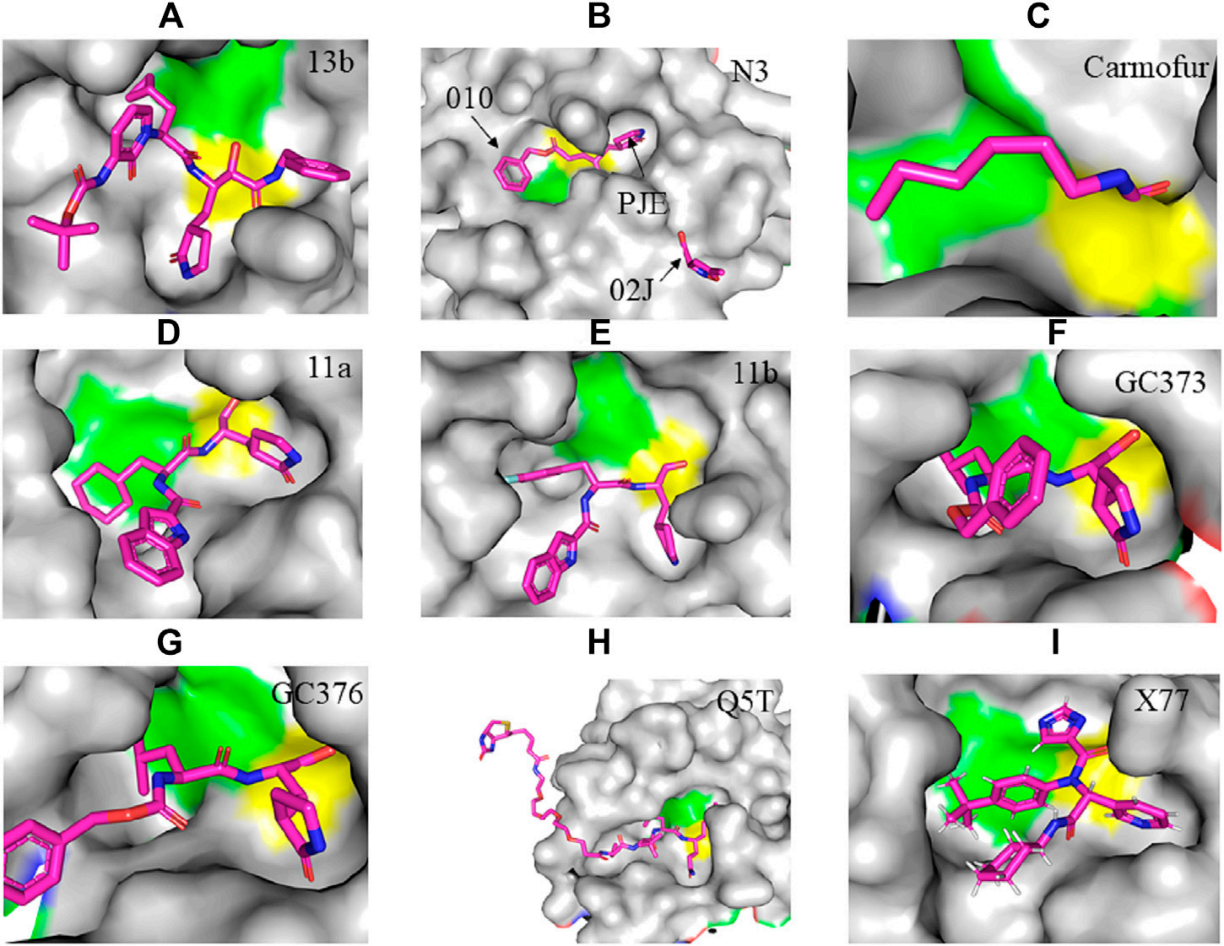
Crystal structure of SARS-CoV-2 M^pro^ in complex with potential inhibitors. **(A)** 13b (PDB entry: 6Y2G, 2.20 Å resolution) **(B)** Michael acceptor N3 (PDB entry: 6LU7, 2.16 Å resolution) **(C)** Carmofur (PDB entry: 7BUY, 1.60 Å resolution) **(D)** 11a (PDB entry: 6LZE, 1.505 Å resolution) **(E)** 11b (PDB entry: 6M0K, 1.504 Å resolution) **(F)** GC373 (PDB entry: 6WTK, 2.00 Å resolution) **(G)** GC376 (PDB entry: 6WTT, 2.15 Å resolution) **(H)** Q5T (PDB entry: 6Z2E, 1.70 Å resolution) and **(I)** X77 (PDB entry: 6W63, 2.10 Å resolution). Residues in the catalytic dyad: His^41^ is highlighted green and Cys^145^ is highlighted yellow. The carbon atoms of each drug are highlighted in magneta.

**FIGURE 3:**
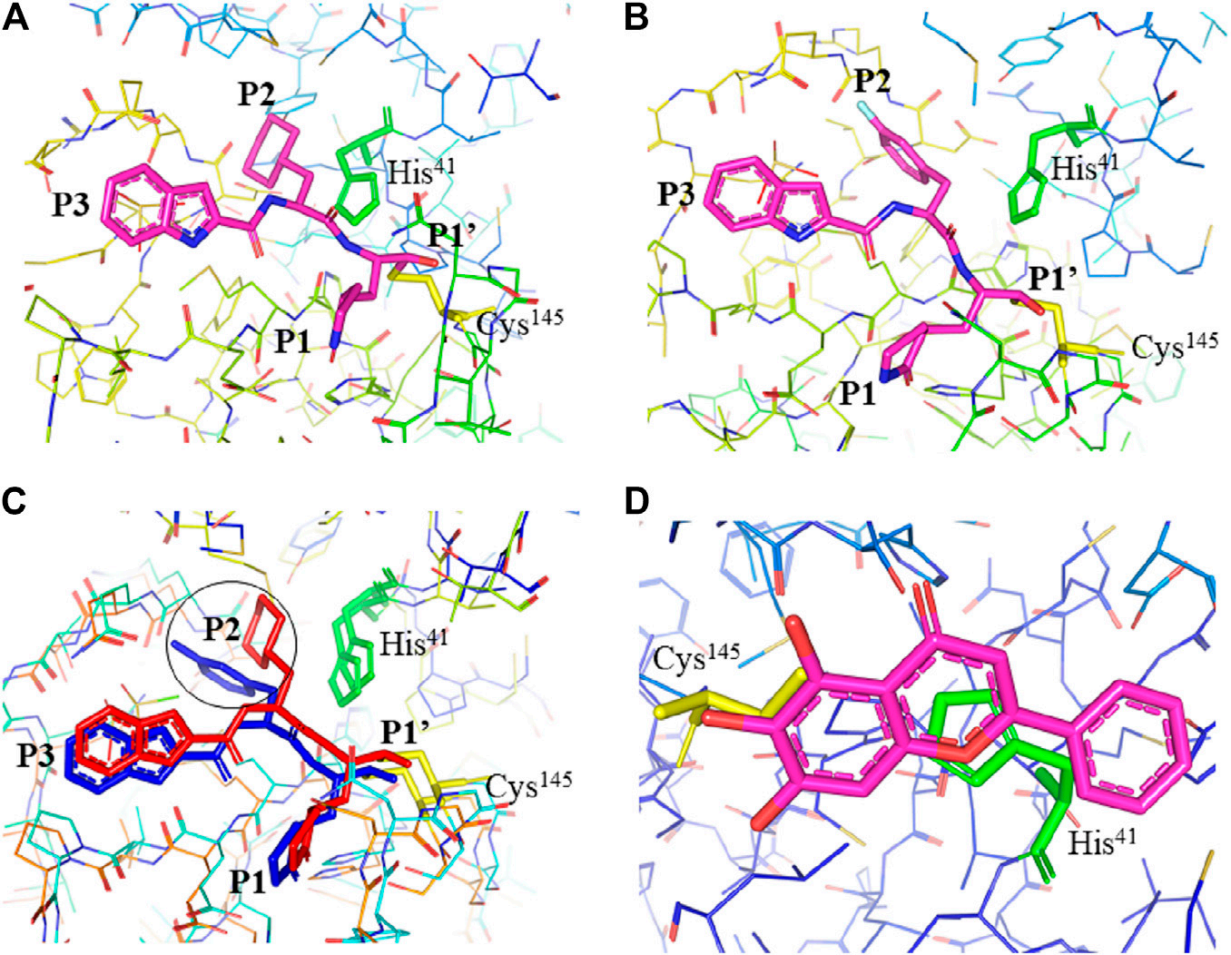
Interaction of 11a, 11b, and baicalein (PDB entry: 6M2N, 2.198 Å resolution) with SARS-CoV-2 M^pro^. **(A)** Interaction of 11a and **(B)** 11b with residues at the subsites of the substrate-binding cleft. The carbon atoms of 11a and 11b are highlighted in magneta. **(C)** Comparison of the difference in the binding modes of 11a (red) and 11b (blue). The main difference at the P2 position is encircled. **(D)** The unique binding of baicalein perfectly inserted in the core position of the substrate-binding pocket where the S1/S2 subsites and the oxyanion loop shielding the active site from a peptide substrate (Su et al., 2020). Baicalein is highlighted in magneta. His^41^ is highlighted green and Cys^145^ is highlighted yellow.

**FIGURE 4:**
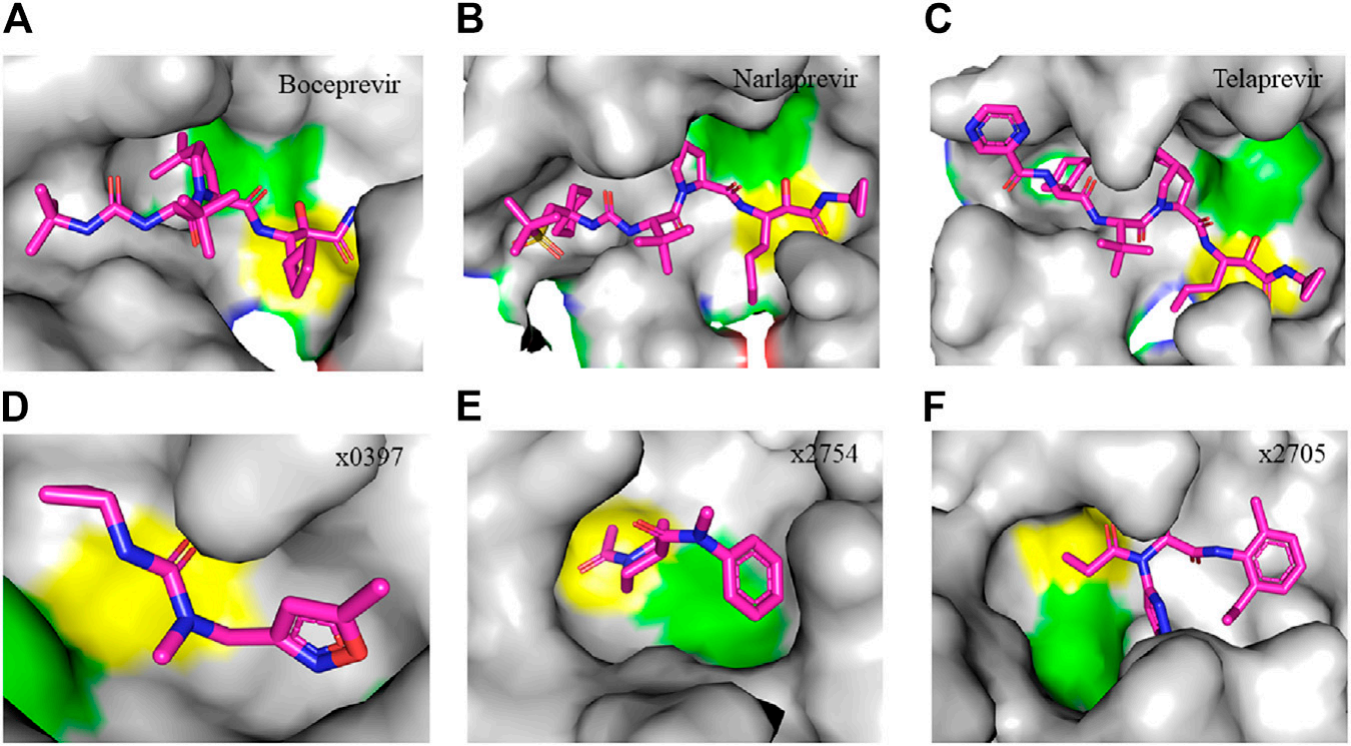
Crystal structure of SARS-CoV-2 M^pro^ in complex with clinically approved antiviral drugs and PanDDA analyzed fragments. **(A)** Boceprevir (PDB entry: 7K40, 1.35 Å resolution) **(B)** narlaprevir (PDB entry: 7JYC, 1.79 Å resolution) **(C)** telaprevir (PDB entry: 7K6D, 1.48 Å resolution) (D) x0397 (PDB entry: 5RGI, 1.57 Å resolution) **(E)** x2754 (PDB entry: 5RHF, 1.76 Å resolution) and **(F)** x2705 (PDB entry: 5RH7, 1.71 Å resolution). Residues in the catalytic dyad: His^41^ is highlighted green and Cys^145^ is highlighted yellow. The carbon atoms of each drug are highlighted in magneta. All structures described in the figures in this paper are solved by X-ray crystallography.

Drugs that specifically bind to and inhibit SARS-CoV-2 M^pro^ could be promising alternatives to fight the pandemic. Gly^143^ of SARS-CoV-2 M^pro^ is reported to be the most attractive residue to form hydrogen bonds with ligands followed by Glu^166^, Cys^145^, and His^163^ (Nguyen et al., 2020). Therefore, determining the crystal structure of viral proteases in a complex with potential inhibitors is vital as it provides a glimpse into designing improved drugs through the modification of the inhibitors according to the structural dynamics (monomer or dimer, narrow or wide, deep or shallow) of the active site in the target enzymes. For example, AG7088 is a potent inhibitor of Rhinoviruses and other Picornaviral 3C-like proteases (3C^pro^), but not for SARS-CoV M^pro^ because the latter is a monomer with only two catalytic domains (Binford et al., 2005; Shie et al., 2005; Kuo et al., 2009) indicating the importance of modifying drugs accordingly, as sequence differences and structural alterations significantly affect the specificity of inhibitors. Additionally, the monomer of M^pro^ is principally considered inactive and therefore the dimer is the best alternative drug target (Grum-Tokars et al., 2008; Pillaiyar et al., 2016). Moreover, designing inhibitors based on their competitive binding to the active site, could help in identifying the best inhibitors. The illustration of the binding of different compounds with SARS-CoV-2 M^pro^ is described in Figures 2–4.

Earlier, Xue et al. (2008) demonstrated the crystal structure of infectious bronchitis virus (IBV) M^pro^ and an active site mutant, His41Ala, SARS-CoV M^pro^ in complex with N-terminal autocleavage substrate, and described the implications for substrate binding and antiviral drug design. Unlike IBV M^pro^, the outer wall of SARS-CoV M^pro^ in the S1 subsite is made up of residues 141 to 143. The S1 and S2 pockets of IBV M^pro^ are comparatively larger than SARS-CoV M^pro^, since Ala^140^ of IBV M^pro^ is away from the active site and its Lys^45^ is about 2 Å away from the S2 subsite. Here the authors suggested that modification of the P3 position of substrates to have a large side chain is a good choice to design substrate-based inhibitors for the main protease of coronaviruses. New inhibitors, N27 and H16, which have relatively large side chains at the P3 position compared to the previously designed inhibitor N3, showed more potent inhibition of SARS-CoV M^pro^ but similar activity with N3 against IBV M^pro^. Moreover, the N3 inhibitor inactivated the M^pro^ of IBV *in vitro* and demonstrated potent antiviral activity against IBV in chicken embryos (Xue et al., 2008). Another study (Xue et al., 2007) reported that the addition of residues at the N terminus, not the C-terminus, of SARS-CoV M^pro^ affects the enzyme activity. Based on the crystal structure of wild type SARS-CoV M^pro^ in complex with Michael receptor N3, it has been proven that the first N terminus residues of the enzyme are vital for keeping the inhibitor binding cleft. These studies laid a concrete foundation for the design of broad-spectrum inhibitors for coronaviruses, including SARS-CoV-2.

The crystal structure of SARS-CoV-2 M^pro^ in complex with different potential drugs has been illustrated. Zhang et al. (2020a) reported the X-ray crystal structure of SARS-CoV-2 M^pro^ in complex with peptidomimetic α-ketoamide inhibitors. The authors modified a previously designed inhibitor (**11r**) by incorporating a P2-P3 amide bond into the pyridone ring (**13a**) with an enhanced half-life in plasma and noticeable tropism to the lung. Moreover, they modified **13a** into a more potent but a narrow-spectrum drug **13b (** Figure 2A **)** by replacing the P2 cyclohexyl moiety with a small cyclopropyl where the drug binds in the shallow substrate-binding pocket at the surface of protomers between domains I and II. Although the improvements (**11r** to **13a**) resulted in a pharmacokinetically better drug, structural modifications negatively affected some inhibitory activities of the drug against SARS-CoV-2 M^pro^ (Mengist et al., 2020; Zhang et al., 2020a) indicating the cautious design of candidate drugs.

In their study, Jin et al. (2020a) (Figure 2B) demonstrated the structure of SARS-CoV-2 M^pro^ in complex with N3. Their results showed that N3 binds inside the pocket of the substrate-binding site. The interaction of N3 with the M^pro^ is in such a way that the Sγ atom of Cys^145^ of protomer A forms a covalent bond with the Cβ atom of the vinyl group. The P1 lactam inserts into the S1 subsite forming a hydrogen bond with His^163^ of protomer A, whereas the side chain of leucine at the P2 site inserts deeply into the hydrophobic S2 subsite. Additionally, the side chain of valine at P3 is solvent-exposed and the side chain of alanine at the P4 side is surrounded by the side chains and main chains of protomer A while the P5 form contacts with Pro^168^ of protomer A and with residues 190–191 at the backbone. Overall, the authors illustrated the specific binding of N3 with the main chain of the substrate-binding pocket through multiple hydrogen bonds and then pharmacokinetically exerting a two-step irreversible inactivation of SARS-CoV-2 M^pro^. Additionally, Jin et al. 2020b also demonstrated that carmofur, an antineoplastic drug, binds on the Cys^145^ catalytic dyad of SARS-CoV-2 M^pro^ (Figure 2C) with promising *in vitro* inhibition of virus replication.

Dai et al., (2020) designed two drugs (**11a** and **11b**) against SARS-CoV-2 M^pro^ and described structural-functional considerations in designing potent inhibitors based on the structure of the substrate-binding site. The two drugs were found to be outstanding main protease inhibitors that can also halt SARS-CoV-2 infection. The crystal structure of the complex showed that the aldehyde group of both drugs covalently bind to Cys^145^ with an *in vivo* auspicious pharmacokinetic property. The antiviral activity of the drugs was maintained by covalent anchoring from the thiol of a cysteine residue in the S1’ subsite of the substrate-binding pocket. In case of **11a (** Figure 2D **)**, the carbon atom of the aldehyde group and Cys^145^ of SARS-CoV-2 M^pro^ form a C–S covalent bond. **11b** exhibits a similar inhibitory binding mode with **11a**, with a small difference probably due to the 3-fluorophenyl group of **11b** at P2, which experiences a downward rotation (Figure 2E). The oxygen atom in the aldehyde group in **11a** stabilizes the conformation of the drug by forming a hydrogen bond with the backbone of Cys^145^ in the S1’ subsite while its (S)-γ-lactam ring at P1 fits in the S1 subsite. The differences in the binding modes of **11a** and **11b** are illustrated in Figures 3A–C. Here, the authors demonstrated the prons and cons of modifying drugs at relevant positions (P1, P2, P3, P4, or P5) through detailed structural-functional explanations.

It has been reported that baicalein exhibited a unique binding mode with SARS-CoV-2 M^pro^ as it does not have direct contact with the 12 amino acids which differed SARS-CoV and SARS-CoV-2 main proteases (Su et al., 2020). The binding of baicalein on the S1 and S2 subsites of the active site is possible through multiple hydrogen bonds between three phenolic hydroxyl groups of the ligand and Leu^141^/Gly^143^ at the main chains in addition to Ser^144^/His^163^ at the side chains. Glu^166^ at the main chain form hydrogen bonding with the carbonyl group whereas the insertion of the free phenyl group into the sub-pocket of S2 was maintained by hydrophobic interactions with Gln^189^, Arg^188^, Met^49^, Cys^44^, and His^41^. Furthermore, the aromatic ring of baicalein forms S- *π* and *π*- *π* interactions with Hsis^41^ and Cys^145^, respectively (Figure 3D **)**.

According to Vuong et al. (2020), GC376 was converted into GC373 upon incubation with SARS-CoV-2 M^pro^ which formed a covalent bond with Cys^145^ (Figure 2F). Accordingly, residues of domain II form contacts supporting drug binding where P2 was inserted into the hydrophobic pocket consisting of His^41^, while the S2 subsite was represented by Met^49^ and Met^165^. His^163^ and Glu^166^ side chains form a hydrogen bond with the glutamine surrogate in the P1 position whereas a hydrophobic interaction was noticed with His^172^ while hydrogen bond connects the backbone amide of Glu^166^ with carbonyl in the P3, suggesting the strong binding capacity of the drugs on the catalytic site of the M^pro^. A simulation study by Jain and Mujwar, (2020) showed that Metocurine, a neuromuscular blocking agent, binds specifically with the substrate-binding cavity of the protease enzyme supported with residues Phe^140^, Leu^141^, Cys^145^, His^163^, His^164^, Met^165^, Glu^166^, Leu^167^, and Pro^168^ repurposing the compound as a safe and effective prospective drug.

The crystal structure of three protomers of SARS-CoV-2 M^pro^ complexed with GC376 (Figure 2G) (Ma et al., 2020) showed unique binding configurations, suggesting the potential candidacy of the compound for COVID-19 treatment. The authors reported that GC376 formed numerous hydrogen bonds with the active site supported with covalent bonds which formed with aldehyde bisulfite warhead and Cys^145^. In another study, the oxygen atoms in the vinyl sulfone group of Q5T (Biotin-PEG (4)-Abu-Tle-Leu-Gln-VS (B-QS1-VS)) form hydrogen bonds with the amide groups of Gly^143^ and Cys^145^ while the catalytic cysteine residue was covalently linked to the Cβ atom of the vinyl group. Although the polar side chains of the P3 form hydrogen bonds with Glu^166^, the authors did not find a well-defined pocket for the P3 moiety (Rut et al., 2021) (Figure 2H). A non-covalent broad-spectrum inhibitor X77 also binds to the substrate-binding cleft of SARS-CoV-2 M^pro^ (Figure 2I).

Clinically approved HCV NS3/4A protease inhibitors (boceprevir, narlaprevir, and telaprevir) showed specific binding on the active site of SARS-CoV-2 M^pro^ secondary to the structural similarity between proteases of the two viruses. Molecular docking revealed that boceprevir formed hydrogen bonding with different residues and hydrophobic interactions with key residues His^41^, Leu^141^, His^164^, Met^165^, Glu^166^, and Asp^187^ through unique binding conformation at the active site (Bafna et al., 2020) (Figures 4A–C). Pan-Dataset Density analysis (PanDDA) of SARS-CoV-2 M^pro^ fragment screening showed that several compounds including x0397, x2754, and x2705 bind on the active site with possible inhibitory activities (Figures 4D–F). Crystallographic and electrophilic fragment analysis of SARS-CoV-2 M^pro^ showed the plasticity of the S1’ subsite indicating an improved design of prospective potent inhibitors. For example, the side-chain movement of catalytic residues Cys^145^ and His^41^ was observed upon binding of Z369936976 compared to Z1129283193. Accordingly, the size and shape of the S1’ subsite were altered, resulting in exceptional binding of the compound to both S1 and S1’ subsites (Douangamath et al., 2020). The details of compounds whose complex X-ray structure with SARS-CoV-2 M^pro^ is solved and reported to have potential subsequent antiviral activity, are described in Table 1.

**TABLE 1:** Details of compounds with their complex structure with SARS-CoV-2 M^pro^ solved, which have potential subsequent antiviral activity.

Name	PDBeChem code	PDB entry	Chemical formula	Molecule name	Chemical structure
N3		6LU7	C35H48N6O8	benzyl (3S,6R,9S,E)-9-isobutyl-6-isopropyl-3-methyl-1-(5-methylisoxazol-3-yl)-1,4,7,10-tetraoxo-12-((2-oxopyrrolidin-3-yl)methyl)-2,5,8,11-tetraazapentadec-13-en-15-oate	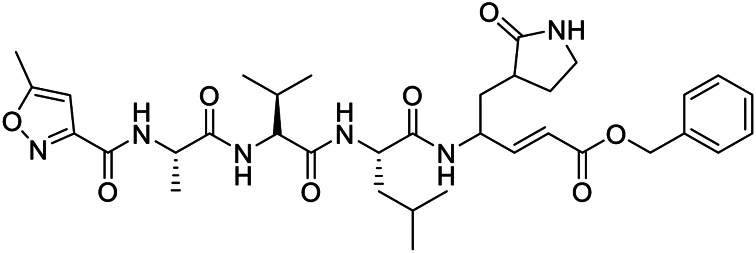
11a	FHR	6LZE	C25H32N4O4	(∼{N}-[(2∼{S})-3-cyclohexyl-1-oxidanylidene-1-[[(2∼{S})-1-oxidanylidene-3-[(3∼{S})-2-oxidanylidenepyrrolidin-3-yl] propan-2-yl] amino]propan-2-yl]-1∼{H}-indole-2-carboxamide	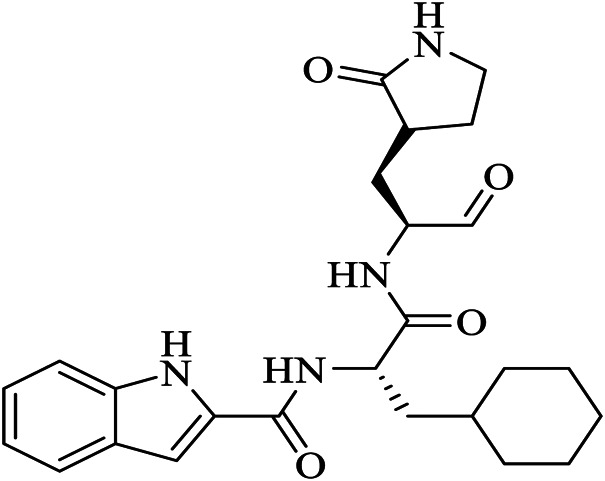
11b	FJC	6M0K	C25H25FN4O4	∼{N}-[(2∼{S})-3-(3-fluorophenyl)-1-oxidanylidene-1-[[(2∼{S})-1-oxidanylidene-3-[(3∼{S})-2-oxidanylidenepyrrolidin-3-yl]propan-2-yl]amino]propan-2-yl]-1∼{H}-indole-2-carboxamide	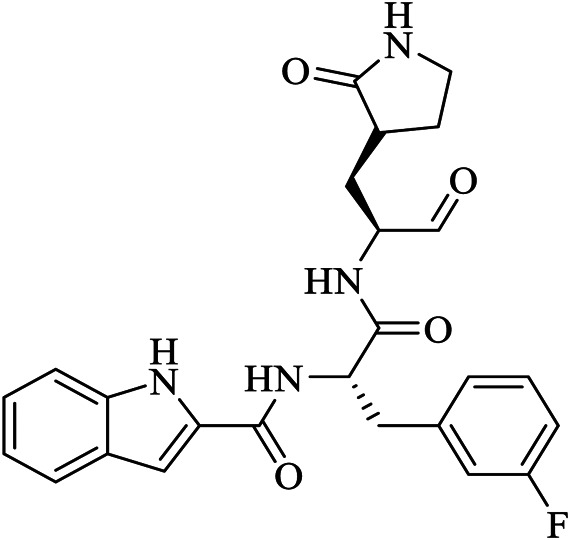
X77	X77	6W63	C27H33N5O2	N-(4-tert-butylphenyl)-N-[(1R)-2-(cyclohexylamino)-2-oxo-1-(yridine-3-yl)ethyl]-1H-imidazole-4-carboxamide	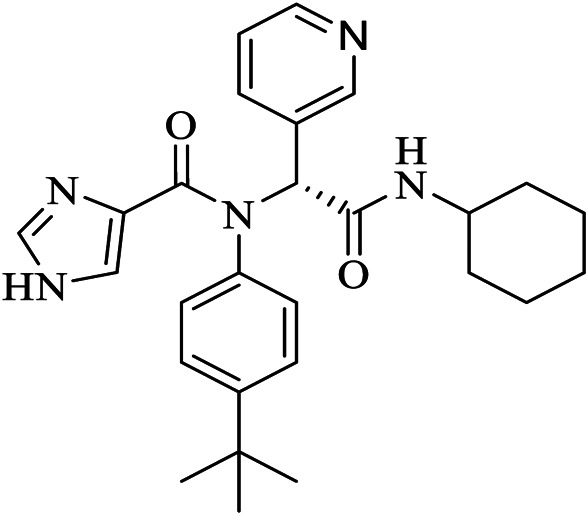
13b	O6K	6Y2G	C31H41N5O7	{tert}-butyl ∼{N}-[1-[(2∼{S})-3-cyclopropyl-1-oxidanylidene-1-[[(2∼{S},3∼{R})-3-oxidanyl-4-oxidanylidene-1-[(3∼{S})-2-oxidanylidenepyrrolidin-3-yl]-4-[(phenylmethyl)amino]butan-2-yl]amino]propan-2-yl]-2-oxidanylidene-pyridin-3-yl]carbamate	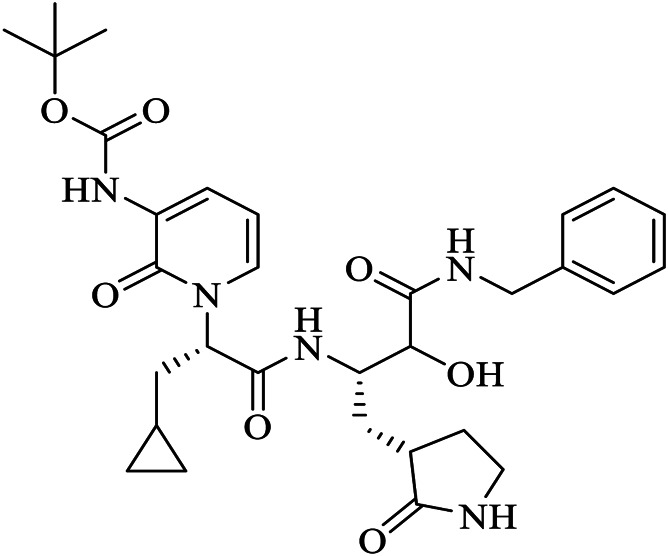
Baicalein	3WL	6M2N	C15H10O5	5,6,7-trihydroxy-2-phenyl-4H-chromen-4-one	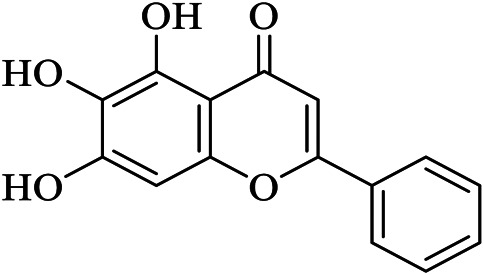
Boceprevir	U5G	7K40	C27H47N5O5	(1R,2S,5S)-N-[(2S,3R)-4-amino-1-cyclobutyl-3-hydroxy-4-oxobutan-2-yl]-3-[N-(tert-butylcarbamoyl)-3-methyl-L-valyl]-6,6-dimethyl-3-azabicyclo[3.1.0]hexane-2-carboxamide	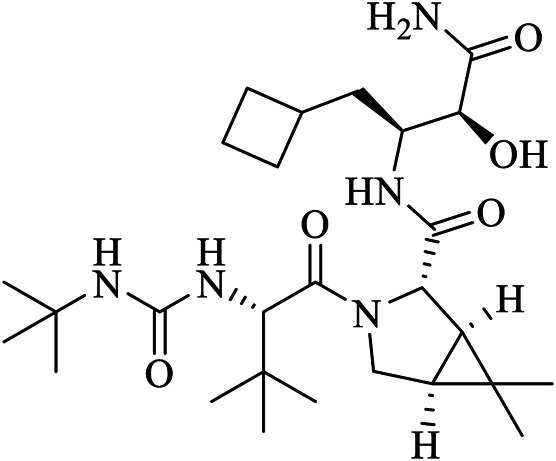
Narlaprevir	NNA	7JYC	C36H63N5O7S	(1R,2S,5S)-3-[N-({1-[(tert-butylsulfonyl)methyl]cyclohexyl}carbamoyl)-3-methyl-L-valyl]-N-{(1S)-1-[(1R)-2-(yclopropyl amino)-1-hydroxy-2-oxoethyl]pentyl}-6,6-dimethyl-3-azabicyclo[3.1.0]hexane-2-carboxamide	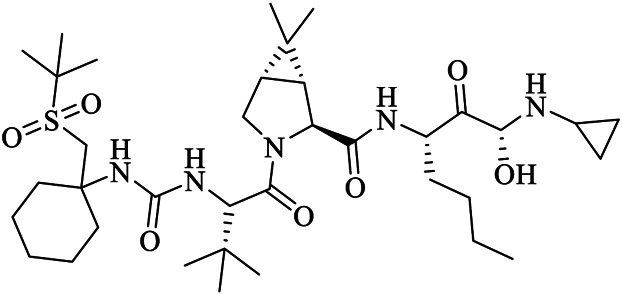
Telaprevir	SV6	7K6D	C36H55N7O6	(1S,3Ar,6As)-2-[(2S)-2-({(2S)-2-cyclohexyl-2-[(pyrazin-2-ylcarbonyl)amino]acetyl}amino)-3,3-dimethylbutanoyl]-N-[(2R,3S)-1-(cyclopropylamino)-2-hydroxy-1-oxohexan-3-yl]octahydrocyclopenta[c]pyrrole-1-carboxamide	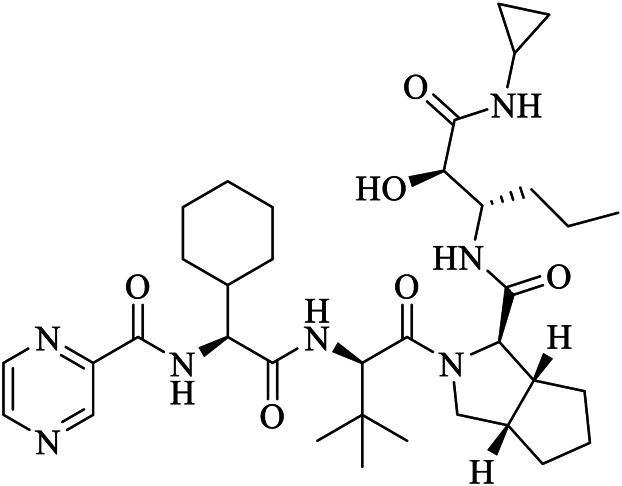
Carmofur	JRY	7BUY	C7H15NO2	hexylcarbamic acid	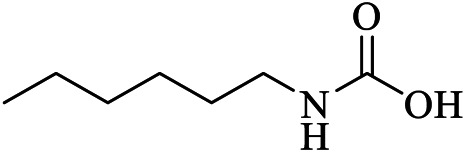
GC373	UED	6WTK	C21H31N3O5	N∼2∼-[(benzyloxy)carbonyl]-N-{(2S)-1-hydroxy-3-[(3S)-2-oxopyrrolidin-3-yl]propan-2-yl}-L-leucinamide	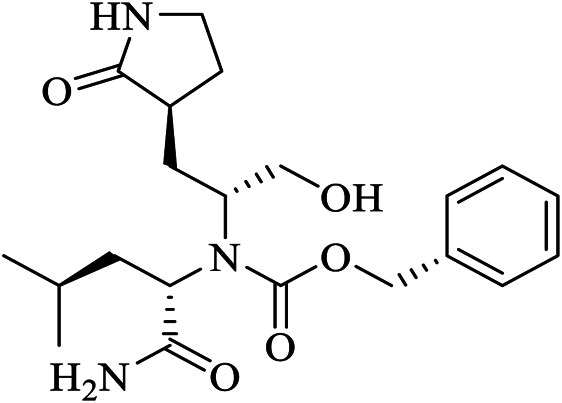
GC376	K36	6WTT	C21H31N3O8S	(1S,2S)-2-({N-[(benzyloxy)carbonyl]-L-leucyl}amino)-1-hydroxy-3-[(3S)-2-oxopyrrolidin-3-yl]propane-1-sulfonic acid	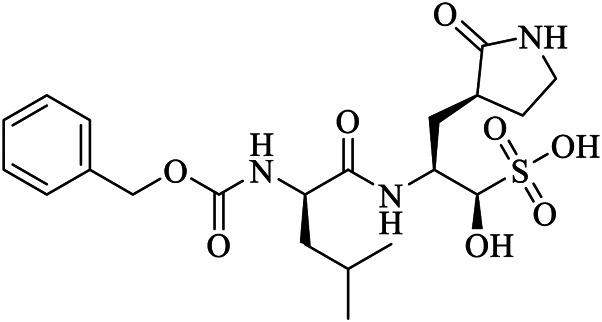
biotin-PEG(4)-Abu-Tle-Leu-Gln-vinylsulfone	Q5T	6Z2E	C44H80N8O13 S2	(4∼{S})-4-[[(2∼{S})-2-[[(2∼{S})-2-[[(2∼{S})-2-[3-[2-[2-[2-[2-[5-[(3∼{a}∼{S},4∼{R},6∼{a}∼{R})-2-oxidanylidene-3,3∼{a},4,6∼{a}-tetrahydro-1∼{H}-thieno[3,4-d]imidazol-4-yl]pentanoylamino]ethoxy]ethoxy]ethoxy]ethoxy]propanoylamino]butanoyl]amino]-3,3-dimethyl-butanoyl]amino]-4-methyl-pentanoyl]amino]-6-methylsulfonyl-hexanamide	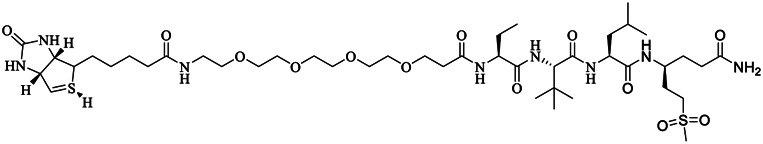
x0397	U0P	5RGI	C10H15N3O2	N'-cyclopropyl-N-methyl-N-[(5-methyl-1,2-oxazol-3-yl)methyl]urea	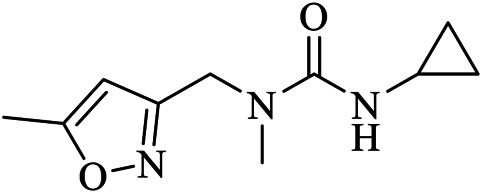
X2754	UPJ	5RHF	C15H20N2O2	1-acetyl-N-methyl-N-phenylpiperidine-4-carboxamide	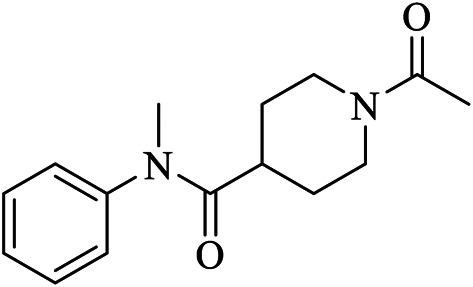
X2705	UJ1	5RH7	C26H33N5O2	N-(5-tert-butyl-1H-pyrazol-3-yl)-N-[(1R)-2-[(2-ethyl-6-methylphenyl)amino]-2-oxo-1-(pyridin-3-yl)ethyl]propanamide	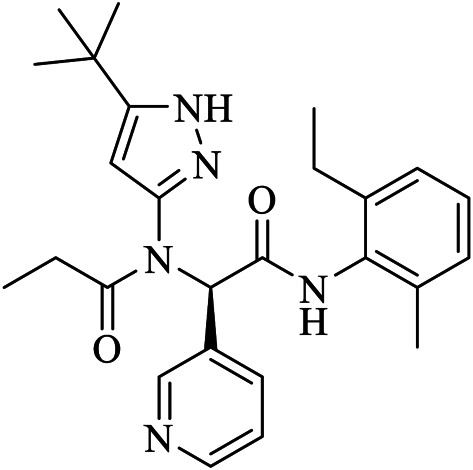

## Potential Inhibitors of SARS-CoV-2 M^pro^

Both *in vitro*/*in vivo*, and *in silico* studies demonstrated that several classes of compounds showed effective binding and inhibition of SARS-CoV-2 M^pro^ (Sharma et al., 2020). In addition to *in vitro* experiments, *in vivo* experiments also illustrated suppression of SARS-CoV-2 infectivity secondary to inhibition of the main protease. For example, significant suppression of multiple coronaviruses by optimized M^pro^ inhibitors was reported in infected mice (Rathnayake et al., 2020). Therefore, pertaining to similar substrate-binding sites across the main proteases of coronaviruses, formulation of broad-spectrum inhibitors could be recommended provided that the structural and functional effect of some key residue differences are well tolerated.

### *In vitro*/*In Vivo* Inhibitors

Alpha-keto amides **11u** and **11r** demonstrated broad-spectrum inhibition of the main proteases of beta coronaviruses and alpha coronaviruses, and the 3C-proteases of enteroviruses in cell culture (Zhang et al., 2020b). Improved compounds **13a** and **13b** (Zhang et al., 2020a) demonstrated specific binding to SARS-CoV-2 M^pro^ with subsequent enzyme inactivation and reduction of viral infectivity. N3 is an irreversible Michael acceptor inhibitor (Yang et al., 2005) which covalently binds with SARS-CoV-2 M^pro^ through Michael reaction, blocking its active site (Griffin, 2020; Jin et al., 2020a). Cell-based assays showed strong antiviral activity of N3 at 10 µM concentration in SARS-CoV-2 infected Vero cells (Jin et al., 2020a). Carmofur has been used to treat several cancers (Nishio et al., 1987; Morimoto and Koh, 2003; Sakamoto et al., 2005) which also demonstrated a clinical potential inhibition of SARS-CoV-2 through targeting M^pro^ (Jin et al., 2020a; Jin et al., 2020b).

Ebselen is an anti-inflammatory, anti-oxidant, and cytoprotective drug which has been studied for treating multiple diseases including bipolar disorders (Singh et al., 2013) and hearing loss (Lynch and Kil, 2009; Kil et al., 2017). This compound showed low cytotoxicity in rats (Renson et al., 1982) whereas whether it is safe for humans is under investigation (Lynch and Kil, 2009; Masaki et al., 2016; Kil et al., 2017). Ebselen specifically binds on the active site of SARS-CoV-2 M^pro^ and showed strong antiviral activity recommended for treating diseases associated with coronaviruses (Jin et al., 2020a). Sies and Parnham (2020) discussed ebselen as a potential drug for COVID-19 with promising inhibition of SARS-CoV-2 M^pro^ provided that the *in vivo* antiviral activity of the drug is determined.

Cinanserin is a well-characterized serotonin antagonist that showed a strong reduction of SARS-CoV replication through inhibiting viral 3CL^pro^ (Chen et al., 2005). This compound demonstrated a moderate inhibitory activity against SARS-CoV-2 M^pro^ (Jin et al., 2020a) suggesting the potential role of cinanserin in preventing coronavirus diseases following targeted modification. Famotidine is an AG protein-coupled receptor antagonist under clinical trial for COVID-19 treatment which showed weak binding affinity to SARS-CoV-2 M^pro^ and only intravenous administration was suggested to be advantageous (Ortega et al., 2020) given that structural modifications could enhance its binding energy and antiviral activity.

Aldehydes are compounds consisting of -CHO as a functional group and carbonyl center (a carbon double bonded to oxygen) where the carbon atom is also bonded to a hydrogen atom or any generic alkyl or side chain R group (alkyl or saturated hydrocarbon). Dai et al., (2020) designed aldehyde-based drugs and reported that compounds **11a** and **11b** showed high anti-SARSCoV-2 M^pro^ activity with inhibition of 100% and 96% at 1 µM, respectively. These compounds specifically bind on the Cys^145^ of the catalytic dyad of M^pro^ and block its activity. Peptide aldehydes also inhibit the main protease of Feline coronavirus (FCoV) (Kim et al., 2015). Among these drugs, GC376 (a prodrug) and GC373 (a drug) specifically bind on the catalytic dyad of recombinant SARS-CoV-2 M^pro^ with potent *in vitro* inhibition at the nano-molar level (Vuong et al., 2020). According to Rathnayake et al. (2020), among compounds tested, **6e** showed more potent antiviral activity in SARS-CoV-2 infected Vero E6 cells, while **7j** showed effective binding with SARS-CoV-2 M^pro^. The authors illustrated effective inhibition of multiple coronaviruses and increased survival of infected mice treated with M^pro^ inhibitors.

Nelfinavir is a protease inhibitor used to treat HIV and is predicted to be a potential inhibitor of SARS-Cov-2 M^pro^ as it showed strong binding affinity to the enzyme (Xu et al., 2020). Further, lopinavir and ritonavir bind to viral main proteases (Nukoolkarn et al., 2008) and have demonstrated effective suppression of the virus through binding and inactivating the M^pro^, as evidenced by effective activity on SARS-CoV-2 patients (Liu and Wang, 2020). Lopinavir and ritonavir also showed a high binding ability to the active pocket of SARS-CoV-2 M^pro^ where Thr^24^, Thr^26^, and Asn^119^ are the key residues important for binding. Furthermore, several commercial medicines including colistin (antibiotic), valrubicin (antitumor), icatibant (indicated for hereditary angioedema), bepotastine (prescribe for rhinitis), caspofungin (antifungal), and perphenazine (antipsychotic) also bind to the protease even with more tolerance to mutation than lopinavir/ritonavir, suggesting possible candidate drugs (Liu and Wang, 2020). Lopinavir and ritonavir were found to have a poor effect in treating COVID-19 pneumonia in addition to their toxic side effects (Wu et al., 2020).

Thirteen potential inhibitors of recombinant SARS-CoV-2 M^pro^ with IC_50_ values ranging from 0.2–23 µM were identified through biochemical high throughput screening. Among them, thimerosal, phenylmercuric acetate, and Evans blue demonstrated the highest inhibitory activity with IC_50_ values below 1 µM (Coelho et al., 2020). Su et al. (2020) reported natural products, baicalin and baicalein (non-covalent, non-peptidomimetic compounds), derived from Chinese traditional medicine as novel inhibitors of the M^pro^. In Vero E6 cells, baicalin and baicalein showed potent antiviral activities with respective IC_50_ values of 6.41 ± 0.95 and 0.94 ± 0.20 µM, indicating a better performance of baicalein over baicalin.

### *In silico* Inhibitors

Computational modeling is an emerging area of research making drug discovery efforts more successful. However, it depends on various factors including protein-ligand geometry, chemical interactions, protonation, hydration, quantum effects, and several other constraints. These complex molecular dynamics computations are expensive and thus molecular docking tools are currently in practice to estimate the binding affinity and stability of protein-ligand interactions (Morris et al., 2009; Cofala et al., 2020). These tools are currently being extensively utilized to discover potential inhibitors that target SARS-CoV-2 M^pro^.

Both new and known antiviral compounds have been studied for their effective binding to the active site of SARS-CoV-2 M^pro^. Among these, a molecular docking study showed that HCV NS3/4A protease inhibitors (sovaprevir, vaniprevir, glecaprevir, boceprevir, simeprevir, paritaprevir, danoprevir, and grazoprevir) bind effectively on SARS-CoV-2 M^pro^ (Bafna et al., 2020) indicating their possible clinical significance. Another study showed that FDA-approved antiviral drugs lopinavir-ritonavir, tipranavir, and raltegravir showed strong, stable, and flexible binding on the active site of SARS-CoV-2 M^pro^ (Kumar et al., 2020c). Four antiviral molecules (Prulifloxacin, Nelfinavir, Tegobuvir, and Bictegravir) were also reported to bind on the active site of SARS-CoV-2 M^pro^ suggesting their ability to block viral protease and thus infection (Li et al., 2020a). Further, fragment-based drug designing identified 47 target compounds of which **#46** showed strong binding potential. Accordingly, the triazole ring binds to the S1 subsite, the covalent fragment of α, β-unsaturated aldehyde binds to S1' subsite, β-lactam ring binds to S2 subsite, and 5,7-dihydroxy chromone binds to S3 subsite of SARS-CoV-2 M^pro^. The Triazole ring forms a H-bond with His^163^, the fragment of α,β-unsaturated aldehyde forms a covalent bond with Cys^145^, aldehyde carbonyl forms a H-bond with His^41^, while the hydroxyl of chromone at position 7 forms a H-bond with Thr^190^ (Tang et al., 2020).

Repurposing existing drugs facilitates the time needed to discover potent compounds for new diseases. Virtual screening of known drugs identified 15 potent inhibitors of SARS-CoV-2 M^pro^ where dipyridamole was the most potent inhibitor followed by candesartan cilexetil, hydroxychloroquine, and chloroquine with respective IC_50_ values of 0.60 ± 0.01, 2.8 ± 0.3, 2.9 ± 0.3, and 3.9 ± 0.2 µM (Li et al., 2020b). Sixty-six FDA-approved drugs demonstrated higher binding scores in a pharmacophore-based drug activity analysis. Based on this, several classes of drugs such as viz. D2 receptor antagonist, HMG-CoA inhibitors, HIV reverse transcriptase and protease inhibitors, anticancer agents, and folate inhibitors presented potential interaction with SARS-CoV-2 M^pro^. Among top-scoring compounds, imatinib showed a promising protease inhibition at 9.823 µM (Balaramnavar et al., 2020). In another *in silico* study, four known drugs (remdesivir, simeprevir, nafamostat, and foretinib) were docked to bind on the catalytic dyad of the main protease. Also, drugs including bromocriptine (a dopamine antagonist), ergotamine (antimigraine), bictegravir (antiviral), antibacterial agents (oxytetracycline, tigecycline, ceftolozane), and immune modulators (vinflunine, vindesine, and topotecan) exhibited effective binding on the active site of the M^pro^ (Chakraborti et al., 2020) showing that the repurposing of several classes of known drugs is crucial to identifying the best drugs for COVID-19 treatment.

More *in silico* studies investigating the potential binding of lead compounds on the M^pro^ of SARS-CoV-2 are emerging. A study (Abel et al., 2020) identified 12 best hits from Super Natural II and Traditional Chinese Medicine databases. Selvaraj et al. (2020) also identified potential compounds from the Traditional Chinese Medicine database interacting with active site residues (His^41^, Gly^143,^ and Cys^145^) of the M^pro^. A ligand and virtual screening study (Ferraz et al., 2020) reported on three approved drugs (glibenclamide, bedaquiline, and miconazole) that effectively bind on the active site of SARS-CoV-2 M^pro^ with possible inhibitory activities. In an attempt to predict potential M^pro^ inhibitors from known antivirals, Kanhed et al., (2020) identified ritonavir, nelfinavir, and saquinavir to be potent M^pro^ inhibitors. Structurally, ritonavir formed hydrogen bonds with Gly^143^ and Cys^145^ with its (thiazoly-5-yl) methylcarbamate of oxygen while the thiazolyl ring forms polar contacts with Thr^25^, Thr^26^, and Leu^27^ of the S1’ subsite. Nelfinavir stabilized its binding with M^pro^ *via* hydrogen bonding with Glu^166^, and with His^41^ and Tyr^54^ in the S2 subsite. Depending on the structure of the pocket, compounds containing oxirane rings are suggested to be good M^pro^ inhibitors (Palese, 2020). Arbutin, terbutaline, barnidipine, tipiracil, and aprepitant were identified as potential hits forming different hydrophilic, hydrophobic, and electrostatic interactions with M^pro^ (Baby et al., 2020). Thioflavonol is a synthetic flavonoid analog that showed a strong binding with the conserved residues in the S1 subsite (Batool et al., 2020).

Fragment-based approaches to identify low molecular weight drugs are also other promising areas of investigation. Gao et al. (2020) repurposed low molecular weight drugs using a fragment-based approach for COVID-19 treatment where the authors identified low molecular weight drugs containing pharmacophores of niacin and hit 1, binding and inhibiting SARS-CoV-2 M^pro^. A niacin derivative, carmofur, showed strong binding and M^pro^ inhibition with a IC_50_ value of 2.8 ± 0.2 µM. Moreover, other low molecular weight analogs of hit 1 including triclabendazole, emedastine, omeprazole, and bendamustine were identified. Carmofur and bendamustine were reported to show potent inhibition whereas omeprazole was suggested for combinational use with another hit 1 analog. Choudhury, (2020) screened 191,678 fragments for their binding ability on the cavity of SARS-CoV-2 M^pro^. The authors then generated new molecules tailored from those fragments which demonstrated strong binding on the adjacent sub-pockets. Finally, 17 molecules with binding abilities were found from which 15 molecules form a stable binding. Luan and Huynh, (2020) merged three-drug fragments (JFM, U0P, and HWH) into B19 which showed a slightly better free binding energy than the native peptide cleaved by the M^pro^.

A 1,3-benzodioxolyl sulfonamide fragment from LASSBio-1945 was identified as a potential inhibitor of SARS-CoV-2 M^pro^ by applying a molecular docking and fragment-based pharmacophore model. The compound exhibited a strong binding energy interacting with residues His^41^, His^163^, and Glu^166^ and potential inhibitory activity with IC_50_ value of 15.97 µM. Here, His^41^ forms hydrophobic interactions and His^163^ donates hydrogen bonds whereas Glu^166^ serves as a hydrogen bond donor or acceptor. A 3-amino-pyridinyl moiety found in several fragments including 1, TRY-UNI-714a760b-6, and EDG-MED-0da5ad92–2 was also found to show effective inhibitory properties with respective IC_50_ values of 24.57 and 53.72 μM (Franco et al., 2020). An amino acid decomposition analysis together with a molecular dynamic simulation was also applied when looking for SARS-CoV-2 M^pro^ inhibitors where hydrogen bonds and hydrophobic interactions were found to hold the complex (Choudhury, 2020). For example, Glu^166^ formed a permanent hydrogen bond with ZINC_252512772 while hydrophobic interactions were observed with His^41^ (Razzaghi-Asl et al., 2020). In addition, histone deacetylase inhibitors (Mamdouh et al., 2020), Cobalt (III) (Kozak et al., 2020), and Copper (II) (Garza-Lopez et al., 2020) are also reported to bind and inhibit SARS-CoV-2 M^pro^. These studies (Garza-Lopez et al., 2020; Kozak et al., 2020) suggested that the positively charged metal ions binding on the negatively charged imidazole ring of stable histidine residues at positions 41,163, 164, and 246, and the thiolate of cysteine residues at positions 44 and 145 could break up the bonds, resulting in inhibition of the M^pro^ activity.

Phytochemicals, extracted from medicinal plants, are now worth studying in the search for inhibitors of SARS-CoV-2 M^pro^. Several phytochemicals including 5,7,30,40-Tetrahydroxy-2’-(3,3-dimethylallyl) isoflavone, Myricitrin, Methylrosmarinate, licoleafol, and amaranthin have been studied to bind and inhibit SARS-CoV-2 M^pro^. Among these, 5,7,30,4’-tetrahydroxy-2’ (3,3- dimethylallyl) is an isoflavone extracted from *Psorothamnus arborescens* which showed high binding affinity, forming strong hydrogen bonds with residues in the catalytic dyad (Cys^145^ and His^45^). Moreover, this extract showed a significant interaction with receptor-binding residues of SARS-CoV-2 M^pro^ even more than the approved drugs (Nelfinavir, Prulifloxacin, and Colistin) (ul Qamar et al., 2020) but investigating the clinical applicability of these drugs is the next area of scrutiny. Another molecular docking study (Shree et al., 2020) reported that six phytochemicals (Withanoside V, Somniferine, Tinocordiside, Vicenin, Isorientin 40-O-glucoside 200-O-phydroxybenzoagte, and Ursolic acid) exhibited strong binding with possible inhibition of SARS-CoV-2 M^pro^. Chikhale et al. (2020) also reported that Asparoside-C, extracted from *Asparagus racemosus*, binds on the substrate-binding pocket of SARS-CoV-2 M^pro^ suggesting its possible inhibitory effect. Ten ligands from olive and four ligands from turmeric exhibited the best lowest binding energies with SARS-CoV-2 M^pro^ (Saif et al., 2020).

A study also showed that different phytochemicals effectively bind on SARS-CoV-2 M^pro^ suggesting their possible medicinal importance (Srivastav et al., 2020). Ursolic acid, carvacrol, and oleanolic acid showed stable and favorable energies resulting in strong binding of these phytochemicals on the active site of the enzyme (Kumar et al., 2020a). Chrysosplenetin is a phytochemical which showed strong binding affinity to the active site of SARS-CoV-2 M^pro^ interacting with residues Leu^141^, Gly^143^, Ser^144^, and Cys^145^ (Ebada et al., 2020). Flavonoids are abundant in plants, fruit, and vegetables. Glycosylated flavonoids were suggested to be good inhibitors where Quercetin-3-O-rhamnoside showed the highest binding affinity. The sugar moiety of these compounds is found to be important for activity as the best compounds have sugar in their target structure (Cherrak et al., 2020). Cucurbitacins from foodstuffs strongly bind on different enzymes of SARS-CoV-2 with cucurbitacin G 2-glucoside and cucurbitacin H showing good drug-likeness properties (Kapoor et al., 2020). Seventeen potent M^pro^ inhibitors were identified from the Marine Natural Product (MNP) library using the pharmacophore model and molecular docking technique. The ligand-enzyme complex at the active site was stabilized by hydrogen bonds with Thr^24^, Ser^46^, Asn^142^, Glu^166^, and Pro^168^ whereas *π*-hydrogen bonds and hydrophobic interactions connect the ligands with His^41^, Gly^143^, and Met^49^, Met^65^, Leu^141^, and Pro^168^, respectively (Gentile et al., 2020) suggesting their potential clinical use. As most studies on phytochemicals report only *in silico* screening results, the actual experimental inhibitory effects are not described requiring future investigation.

## Discussion and Future Perspectives

This review discusses the structure-based design of inhibitors targeting SARS-CoV-2 M^pro^ and highlights the antiviral activity of potential candidate drugs of COVID-19. Especially in this time of urgent therapeutic need to treat COIVID-19, pharmaceutical repurposing and structure-based designing of drugs play significant roles in the fast discovery of potent drugs which in turn, apart from reaching the treatment needs of the community, also saves time and resources. Provided this, the structure-based design of drugs requires producing high-quality structures. The desired inhibitors should also have high binding specificity with the target (to minimize off-target binding), be competitive (increased affinity) and flexible (increased efficacy), easy for administration, and have an acceptable plasma half-life.

While the structure-based design of drugs is a robust approach, translation of the structural information into practice is another challenge. Moreover, X-ray structures present a static state of proteins which affects the design of effective drugs as the static structures may not be the most representative conformations of active enzymes. The designed drug might have other clinical shortcomings like high toxicity, teratogenicity, quick metabolism, inability to reach the target site, quick clearance, instability, is difficult to synthesize, and be costly to the general public (Verlinde and Hol, 1994; Craig and Eakin, 2000). This indicates that the successful design of a drug which specifically binds to the target does not mean success, rather, that the structure-based drug design needs to be done cautiously. Another challenge is designing *de novo* drugs using unliganded target proteins alone, nevertheless, computational approaches have significantly overcome this challenge. However, scoring is considered a serious problem since a large number of potential ligands are generated during molecular docking (Kuntz, 1992; Craig and Eakin, 2000). Another important issue in structure-based drug design is the optimization of the compounds based on the pharmacophore requirements of the M^pro^. In a study, it was reported that only cinanserin showed best binding affinity and inhibitory activity after optimizing 220 compounds (Stoddard et al., 2020).

*In silico* studies are important for a better understanding of the M^pro^ structure and function which is a key factor when designing drugs. Moreover, computational drug design methods have an indispensable role in predicting the best drug, among others. Studies focusing on the *in silico* design of potent drugs targeting SARS-CoV-2 M^pro^ are increasing steadily; however, the clinical use of these desired drugs is questionable, corresponding to the possible limitations of passing clinical trials. Recently, a structural simulation study (Ahamad et al., 2020) screened three malaria-box compounds (MB-241, MB-250, and Mb-266) as the best lead drugs binding on SARS-CoV-2 M^pro^; however, whether these compounds have experimental and/or clinical inhibitory activities is unknown. The development of drugs with broad-spectrum antiviral activity is considered a long purposed goal in drug discovery (Maurya et al., 2020). Therefore, using previously approved broad-spectrum drugs after appropriate improvements in design and potency could be an alternative solution during urgent times. In this regard, Zhang and colleagues (Zhang et al., 2020a) designed an improved peptidomimetic α-ketoamide inhibitor (**13a**) from a previously designed broad-spectrum drug (**11r**) (Zhang et al., 2020b). While **13a** is pharmacokinetically improved, some inhibitory activities of **11r** were lost. Further, they modified **13a** into a more potent drug **13b** with compromised broad-spectrum activity, while removal of the Boc group in **14b** inactivated the drug which provides a big lesson for curious design and/or improvement of drugs.

A study by Jin et al. (2020a) found promising cell-based inhibitory activity of screened drugs. The authors found that ebselen and N3 exhibit the strongest cell-based inhibitory activity against M^pro^. Although ebselen, N3, carmofur, and PX-12 bind on the catalytic dyad of M^pro^, carmofur, and PX-12 modified its structure completely. It is noted that the mechanism of covalent modification of M^pro^ by carmofur is different from N3 where N3 modifies Cys^145^ by adding a vinyl group (Jin et al., 2020a). Further, unlike N3 which occupies all four subsites, carmofur is restricted only at the S2 subsite (Yang et al., 2005; Jin et al. 2020b) which also showed a promising lead drug to treat COVID-19 as it inhibits viral replication in cells. In-house designed drugs (**11a** and **11b**) bind on the M^pro^ and inhibit SARS-CoV-2 infectivity. Here the authors designed the inhibitors in such a way that the aldehyde group in the P1 serves as a new warhead to bind covalently with Cys^145^ while the indole group was added in the P3 to form a hydrogen bond with Ser^4^ to enhance its drug-likeness properties. At P2 position, **11a** has cyclohexyl while 3-fluorophenyl is in **11b** which makes **11a** pharmacokinetically better (Dai et al., 2020) (Figure 3). Aldehyde based drugs GC373 and GC376 were also pharmacokinetically effective to inhibit SARS-CoV-2 infection (Vuong et al., 2020). Baicalein also showed unique binding on S1 and S2 subsites of the catalytic dyad with promising *in vitro* inhibition at a IC_50_ value below 1 µM (Su et al., 2020).

GC376 was developed to treat Feline infectious peritonitis and showed potent antiviral activity against MERS-CoV, FIPV, and the norovirus (Kim et al., 2012; Kim et al., 2016; Pedersen et al., 2018). Boceprevir, GC376, and calpain inhibitors (II and XII) inhibit replication of the SARS-CoV-2 virus *via* targeting M^pro^ in the cell culture, with EC_50_ values of 0.49–3.37 µM at acceptable cell cytotoxicity. HCV NS3/4A serine protease inhibitors (boceprevir and narlaprevir) strongly bind and inhibit SARS-CoV-2 M^pro^, with IC_50_ values of 4.13 and 4.73 μM, respectively. Among the screened drugs, GC376 was the most potent M^pro^ inhibitor (IC_50_ = 0.03 μM) while an anti-Rhinovirus drug (rupintrivir) failed to inhibit SARS-CoV-2 M^pro^. Unlike HCV serine protease and SARS-CoV-2 cysteine protease M^pro^, boceprevir and narlaprevir did not inhibit enterovirus A71 2A and 3C proteases and all four drugs (boceprevir, GC376, calpain inhibitor II and calpain inhibitor XII) did not inhibit the unrelated influenza virus H1N1 because of their specificity (Ma et al., 2020), suggesting that broad-spectrum viral protease inhibitors should be clinically investigated before using them. Additionally, boceprevir and GC376 were found to effectively bind and inhibit SARS-CoV-2 M^pro^ (Fu et al., 2020).

HIV protease inhibitors lopinavir and ritonavir are reported to bind and inhibit SARS-CoV (Nukoolkarn et al., 2008) and SARS-CoV-2 M^pro^ with promising antiviral activities (Liu and Wang, 2020). But a contradicting report by Ma et al. (2020) showed that the two drugs failed to inhibit SARS-CoV-2 M^pro^. Similarly, these drugs demonstrated lack of efficacy in a clinical trial of severe COVID-19 adult patients (Cao et al., 2020) with unacceptable toxicity in treating COVID-19 related pneumonia (Wu et al., 2020). This indicates that promising inhibitory activity of drugs either *in silico* or *in vivo* studies does not guarantee clinical efficacy corresponding to complex pharmacodynamics in the human body.

Remdesivir has been approved for COVID-19 treatment in the USA and many vaccine trials are at their last phase while the Pfizer vaccine has been approved for emergency use in some countries (at the time of writing this article). It has been noted that many small steps have been made in discovering clinically applicable drugs to treat COVID-19 (Erlanson, 2020). It is anticipated that vaccines and antibody-based drugs will be discovered before small molecules. However, vaccines might not be 100% effective and antibodies could have immunopathological consequences. Therefore, looking for putative drugs targeting SARS-CoV-2 M^pro^ is necessary. However, there are many challenges in designing drugs that target the proteases of coronaviruses due to poor pharmacokinetic properties of peptidomimetic/high molecular weight compounds and low inhibitory potential of non-peptidomimetic/low molecular weight compounds (Turk, 2006; Drag and Salvesen, 2010). Based on their inhibitory potency and selectivity, focusing on high molecular weight compounds over low molecular weight compounds has been advantageous; however, their drug-likeness property is questionable. On the other hand, 11 residues’ long peptide (WWTWTPFHLLV), showed a strong binding affinity compared to α-keto amide inhibitors with a suggested better inhibitory activity over small molecules (Rossetto and Zhou, 2020). Amin et al., 2020) analyzed the drug-likeness properties of recently reported SARS-CoV-2 M^pro^ inhibitors. The authors reported that only baicalein, disulfiram, carmofur, ebselen, tideglusib, shikonin, and PX-12 passed the drug-likeness evaluation.

A lot has been learned from previous structure-based drug design studies which could help prospective studies succeed fast in discovering effective antivirals for COVID-19 targeting the M^pro^. Accordingly, atomistic-level mechanisms of peptide cleavage and pharmacophore requirements of the M^pro^, stability of inhibitor-enzyme complex similar to the native peptide, plasticity of the active site of M^pro^, the occurrence of mutations at the domains and/or the active site affecting the pocket, and the size and accommodation capacity of the subsites should be considered when designing new drugs or modifying previously known broad-spectrum drugs. This means that the optimization of the inhibitor-enzyme complex is ultimately important. As a significant number of studies solely report the binding affinity and energy of compounds towards the substrate-binding cleft of the M^pro^, improvements considering the abovementioned points should considered in the future. Future, structure-based drug design studies should comprehensively consider potency, selectivity, and drug-likeness properties of the candidate drugs in addition to optimizing their binding ability on the active site. Since low molecular weight compounds and non-peptidomimetic drugs have better drug-likeness properties over their counterparts (Amin et al., 2020), fragment-based drug design strategies should be considered to enhance the potency of these compounds. Drug repurposing is also crucial in urgent times. In this regard, structure-based drug repurposing studies need to determine the dynamics of molecules targeting the M^pro^ (Durdagi et al., 2020). Such strategies may facilitate the efforts of discovering clinically applicable potent drugs for COVID-19.

Several drug targets are available to treat diseases caused by coronaviruses. The essential functions of SARS-CoV-2 M^pro^ in the viral life cycle with a conserved active site and structural suitability of its substrate-binding site for potent drugs, recognize it to be a promising drug target for treating COVID-19. The structural-functional reports so far presented strong pieces of evidence showing the binding specificity and inhibitory roles of compounds against the M^pro^ that could subsequently control SARS-CoV-2 infection. Taken together, their results provide a strong base to design further improved drugs with either limited or broad-spectrum activities with determined potency and pharmacokinetic profiles. Although significant efforts have been made in the search for potent drugs that inhibit SARS-CoV-2 M^pro^, longitudinal studies on the therapeutic safety and efficacy of candidate drugs are still limited, ongoing, or not yet disseminated.

## Conclusion

The main protease of coronaviruses is relatively conserved (Hayden et al., 2003; Yang et al., 2005; Xue et al., 2007; Kim et al., 2016; Stoermer, 2020; Ullrich and Nitsche, 2020) and is what most drug repurposing studies are focusing on. However, mutations at the substrate-binding site and/or other sites due to viral evolution could potentially affect the structure of the M^pro^ substrate-binding pocket. For example, surface loops and helical domains III are variable across different M^pros^ (Jin et al., 2020a) which affect the conformation of the active site (Xue et al., 2007). Moreover, a mutagenesis study depicted that some specific mutations cause major changes on the structure of the protein (Wolfe et al., 2020). Further, some plasticity is reported on the active site of SARs-CoV-2 M^pro^ compared to SARS-CoV M^pro^ (Palese, 2020) which may hinder the design of broad-spectrum drugs. Therefore, updated designs of potential inhibitors that can suitably bind with the active site of the enzyme are ultimately necessary. Similarly, caution should be taken when modifying broad-spectrum inhibitors as the modification could affect the inhibitory activity of some drugs (for example, **13a** lost its broad-spectrum activity upon improved into **13b** (Zhang et al., 2020a)) as designing potent individual drugs for every virus strain is a resource, technical and also time-demanding. Considering the size of functional groups while designing improved drugs is also crucial as it affects the binding modes of drugs on the catalytic dyad of M^pro^ (Dai et al., 2020). Several potential classes of drugs are effective against SARS-CoV-2 M^pro^. Among these, α-ketoamide inhibitors, peptide-based inhibitors, anilid-based inhibitors, drugs from Chinese traditional medicine, phytochemicals, and indole lactam-based inhibitors are amongst the famous drug classes studied well. Although remdesivir is currently approved by the USA-FDA to treat COVID-19 patients, its clinical efficacy remains debatable. Therefore, improved, well-designed, potent, and structurally and pharmacokinetically effective drugs are urgently needed. Further investigations should focus on validating and finalizing effective drugs for COVID-19 beyond preliminary *in silico* and *in vivo* screening.
